# Community-acquired urinary tract infections caused by *Burkholderia* *cepacia* complex in patients with no underlying risk factor

**DOI:** 10.1099/jmmcr.0.005081

**Published:** 2017-01-31

**Authors:** Laila Nimri, Mamuno Sulaiman, Osama Bani Hani

**Affiliations:** ^1^​ Department of Laboratory Medical Sciences, Jordan University of Science and Technology, Irbid, Jordan; ^2^​ Department of General and Pediatric Surgery, Jordan University of Science and Technology, Irbid, Jordan

**Keywords:** *Burkholderia cepacia*, community-acquired, antimicrobial susceptibility pattern, urinary tract infections

## Abstract

**Introduction.** Urinary tract infections (UTIs) remain common infections diagnosed in outpatients as well as hospitalized patients. Community-acquired UTIs are generally caused by *Escherichia coli* and other members of the family *Enterobacteriaceae.* *Burkholderia* *cepacia* is an opportunistic pathogen mainly affecting immunocompromised and hospitalized patients, particularly those who have received prior broad-spectrum antibacterial therapy.

**Case presentation.** Urine samples were collected from 157 outpatients clinically diagnosed with UTI and from 100 healthy control subjects. Samples were cultured on differential media and non-motile lactose-non-fermentors were identified via the Remel RapID ONE system. The isolates were tested by the disc diffusion method against 17 antimicrobial agents. *Burkholderia* was isolated as a single organism from four patients having uncomplicated infections, and one from recurrent infection. None of these patients had an underlying risk factor for this pathogen. Identification of these isolates by the Remel-RapID ONE system was confirmed by *recA* gene amplification. The four isolates were resistant to lincomycin, nalidixic acid, oxacillin and penicillin G. These cases received monotherapy of oral co-trimoxazole.

**Conclusions**. Our findings alert urologists and diagnostic laboratories to the potential of *B.* *cepacia* complex infections in similar cases, and that this bacterium should not be ruled out.

## Abbreviations

CA-UTI, community-acquired UTI; CF, cystic fibrosis; UTI, urinary tract infection.

## Introduction

Uncomplicated urinary tract infections (UTIs) are among the most frequently encountered infections in the outpatient setting [1]. Urine is normally sterile, but intestinal bacteria originating from the anus may gain ascending entry through the urethra or rarely from the bloodstream and cause an infection in the urinary system [2].

The diagnosis of UTI is made based on the clinical picture of illness and urine culture. Most UTIs are uncomplicated, and empirical treatment may be initiated for those patients without the benefit of urine culture [3]. Community-acquired infections are often distinguished from nosocomial, or hospital-acquired, diseases by the types of organisms that affect hospitalized patients. Patients with hospital-acquired UTIs have more comorbidities, and recurrent UTI, and have previously received antibiotics more often than patients with community-acquired (CA)-UTIs [4]. UTI caused by *Burkholderia* *cepacia* was reported after renal transplantation [5], and in recurrent UTIs complete anatomical evaluation was recommended in such cases after renal transplant [6]. *B. cepacia* may also be a causative pathogen for nosocomial UTI in paediatric patients with predisposing factors [7].

*Escherichia coli* remains the predominant uropathogen (80 %) isolated in uncomplicated acute CA-UTI [2]. However, the common pathogens traditionally associated with UTIs are changing many of their features, particularly because of antimicrobial resistance. In addition, complicated UTI has a more diverse aetiology than uncomplicated UTI, and organisms that rarely cause disease in healthy patients can cause significant disease in hosts with anatomical, metabolic or immunological underlying disease [2].

Knowledge of the uropathogens and their antibacterial susceptibility, which may vary with time, is important for treatment. In many clinical laboratories, urine cultures account for 24–40 % of submitted cultures; and 80 % of these urine cultures are submitted from the outpatient setting [8]. Rates of antibiotic resistance have considerably changed, and consequently the empirical treatment of UTI requires constant updating based on the antimicrobial susceptibility of the main uropathogens of the area, or country [6, 9].

This case study reports on CA-UTI caused by members of the *B.* *cepacia* complex in otherwise healthy individuals and the isolates' antibiotic resistance patterns.

## Case report

Urine samples were collected from 157 symptomatic outpatients visiting urology clinics who were clinically diagnosed with UTI and from 100 healthy individuals who did not report any signs or symptoms of UTI in the past year and were willing to participate.

The study protocol was approved by the University Institutional Review Board (IRB). All subjects signed an informed consent form before samples were collected. A structured questionnaire was filled in by each patient, and for control subjects by a trained investigator. The questionnaire included demographic data such as sex and age and questions regarding the infection, clinical data and medication if any.

Urine samples were cultured on selective media for Gram-negative bacteria that were incubated at 37 °C for 48 h. Cultures with a bacterial count of ≥10^3^ c.f.u. per millilitre of urine were considered positive.

Four of the non-motile, lactose-non-fermenting isolates were identified biochemically as *B. cepacia* using the Remel-RapID ONE System (Thermo Scientific) based on the ERIC electronic code compendium (http://www.remel.com/eric/) designed to work exclusively with this system. Identification of the four isolates as members of the *B. cepacia* complex was confirmed by amplifying the *B. cepacia* complex *recA* gene (1040 bp) using primers BCR1 and BCR2 [10]. LB broth (Bioscience) was used for the storage of stock cultures of selected isolates.

This bacterium was isolated as a single organism from two males and two females (age range 28–45 years). Three isolates were from patients having uncomplicated infections, and one isolate was from a female patient having recurrent infections. The most common symptoms reported by patients included urgency to urinate, frequency, discomfort and pain, typically in the lower back and abdominal area, or when urinating.

The antibiotic susceptibility of the four *B. cepacia* complex isolates was assessed *in vitro* to 17 antibiotics by the disc diffusion method on Mueller-Hinton agar (Table 1). All four *B.* *cepacia* isolates were resistant to lincomycin, nalidixic acid, oxacillin and penicillin G. Three were resistant to ampicillin and cefixime, two were resistant to tetracycline, while only one was resistant to amoxicillin, azithromycin, cefotaxime, piperacillin and trimethoprim-sulfamethoxazole. However, all four isolates were susceptible to ceftazidime, ciprofloxacin, gentamicin, imipenem and levofloxacin.

**Table 1.** Antimicrobial susceptibility testing of four *B. cepacia* complex isolates, with the zone of inhibition (mm) as defined by CLSI [21] R, resistant; I, intermediate; S, susceptible.

Antibiotic	Symbol/ disc potency (µg mg^−1^)	108P *B. cepacia* (mm)	078P *B. cepacia* (mm)	087P *B. cepacia* (mm)	047P *B. cepacia* (mm)
Amoxicillin	AMC-30	(3) I	(8) R	(4) S	(5) S
Ampicillin	AM-10	(8) R	(8) R	(2) S	(6) R
Azithromycin	AZM-15	(2) I	(1) S	(1) S	(6) R
Cefixime	CFM-5	(3) I	(7) R	(8) R	(9) R
Cefotaxime	CTX-30	(26) S	(10) R	(24) I	(30) S
Ceftazidime	CAZ-30	(10) S	(28) S	(29) S	(26) S
Ciprofloxacin	CIP-5	(36) S	(30) S	(34) S	(35) S
Gentamicin	CN-10	(1) S	(23) S	(30) S	(1) S
Imipenem	IPM-10	(22) S	(29) S	(30) S	(4) S
Levofloxacin	LEV-5	(36) S	(36) S	(34) S	(30) S
Lincomycin	L-2	(0) R	(0) R	(0) R	(0) R
Nalidixic acid	NA-30	(2) R	(11) R	(2) R	(7) R
Oxacillin	OX-1	(0) R	(0) R	(0) R	(0) R
Penicillin G	P-10 I.U.	(6) R	(8) R	(8) R	(8) R
Piperacillin	PRL-100	(27) S	(28) S	(28) S	(11) R
Tetracycline	TE-30	(0) R	(22) S	(22) S	(8) R
Trimethoprim-sulfamethoxazole	SXT-25	(3) S	(1) S	(1) S	(6) R*

*Isolate from female, recurrent infection.

## Treatment

Treatment consisted of oral co-trimoxazole twice daily for 5–7 days; the dose was weight-dependent.

## Discussion

Uncomplicated CA-UTIs and in hospitalized patients are extremely common infections [1, 11]. In the current study, *B.* *cepacia* complex isolates were among the bacterial species recovered from four outpatients with uncomplicated infections; these patients had no underlying risk factor and no history of recurrent UTI. There was a strong connection to the infection because this bacterium was isolated as a single organism in these patients. Infections with this species might often be misdiagnosed in clinical laboratories because the identification of suspected *B.* *cepacia* (formerly *Pseudomonas cepacia*) isolates is performed using a combination of selective media, conventional biochemical analysis, commercial test systems and PCR-based assays if available [12]. These tests are not routinely used in diagnostic laboratories and several laboratories have experienced difficulty in identifying this bacterium. The *B.* *cepacia* complex is an important nosocomial pathogen in patients, particularly those who have received prior broad-spectrum antibacterial therapy [13]. However, *Pseudomonas cepacia* was first reported in renal calculi in non-immunocompromised patients [14].

An earlier study conducted in Jordan reported a high mortality rate in a nosocomial outbreak caused by *B. cepacia* in patients suffering from diseases other than cystic fibrosis (CF) (bacteraemia or respiratory colonization) [15].

*B. cepacia* is one of the most antimicrobial-resistant organisms, with high intrinsic resistance encountered in the clinical laboratory; and such infections can be very difficult to treat and result in death in some cases [13]. All four urinary *B. cepacia* complex isolates recovered in our study showed resistance or intermediate susceptibility to one or more of the antimicrobial agents. *B. cepacia* complex strains are multidrug-resistant due to innate and acquired mechanisms of resistance [16]. One of the isolates was resistant to trimethoprim-sulfamethoxazole (Table 1). *B. cepacia* is often susceptible to trimethoprim-sulfamethoxazole, but emerging resistance to these antimicrobial agents is of increasing clinical concern, especially among CF patients with *B. cepacia* complex respiratory infection, where only 5 % of over 2600 strains tested were susceptible to this agent [17]. However, the susceptibility profiles of strains from CF patients may differ from those noted in strains from other patients because presumably CF patients receive multiple courses of oral, intravenous and aerosolized antibiotics [18].

Resistance to trimethoprim is mediated by production of dyhydrofolate reductase or acquisition of outer membrane antibiotic efflux pumps that confer cross resistance to chloramphenicol and fluoroquinolones [19]. For serious infection with trimethoprim-sulfamethoxazole-resistant strains or sulfa drug allergy, combination therapy guided by *in vitro* susceptibility results should be given.

In most UTI cases, empirical treatment without the benefit of a pre-therapy urine culture is used. Most clinicians are not aware of *B. cepacia* as a potential uropathogen. The antimicrobial use, whether appropriate or inappropriate, is associated with the selection for antimicrobial-resistant organisms colonizing or infecting the urinary tract. Thus, infections caused by antimicrobial-resistant organisms are associated with higher rates of treatment failure [20]. Therefore, knowledge of the antimicrobial susceptibility profile of uropathogens causing uncomplicated CA-UTIs should guide therapeutic decisions [1].

In conclusion, our finding of *B. cepacia* complex infections in four outpatients with no underlying risk factor alerts clinical diagnostic laboratories to the potential presence of this significant pathogen and to include its identification in similar cases. In addition, the multiresistance of most isolates to several tested antimicrobial agents should guide therapeutic decisions.
